# Supplementary material for: Control of mouse limb initiation and antero-posterior patterning by Meis transcription factors
Source: Nat Commun. 2021 May 25;12:3086. doi: 10.1038/s41467-021-23373-9 (PMC8149412; doi:10.1038/s41467-021-23373-9)
Supplement: Supplementary file 1 — Supplementary Information [file 41467_2021_23373_MOESM1_ESM.pdf]

Supplementary Figures

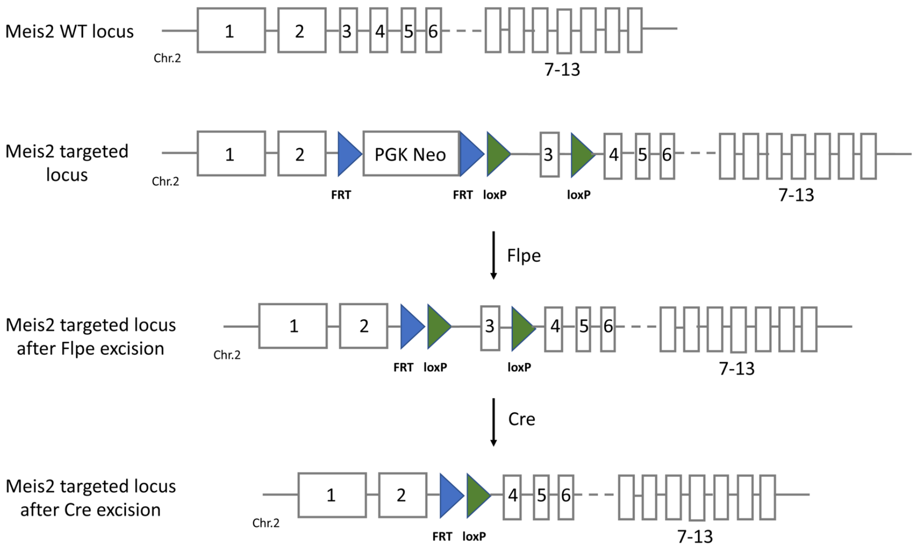

Supplementary Fig 1. Meis2 targeting strategy. Exon3 was flanked by loxP sites resulting in its deletion in the presence of Cre recombinase.

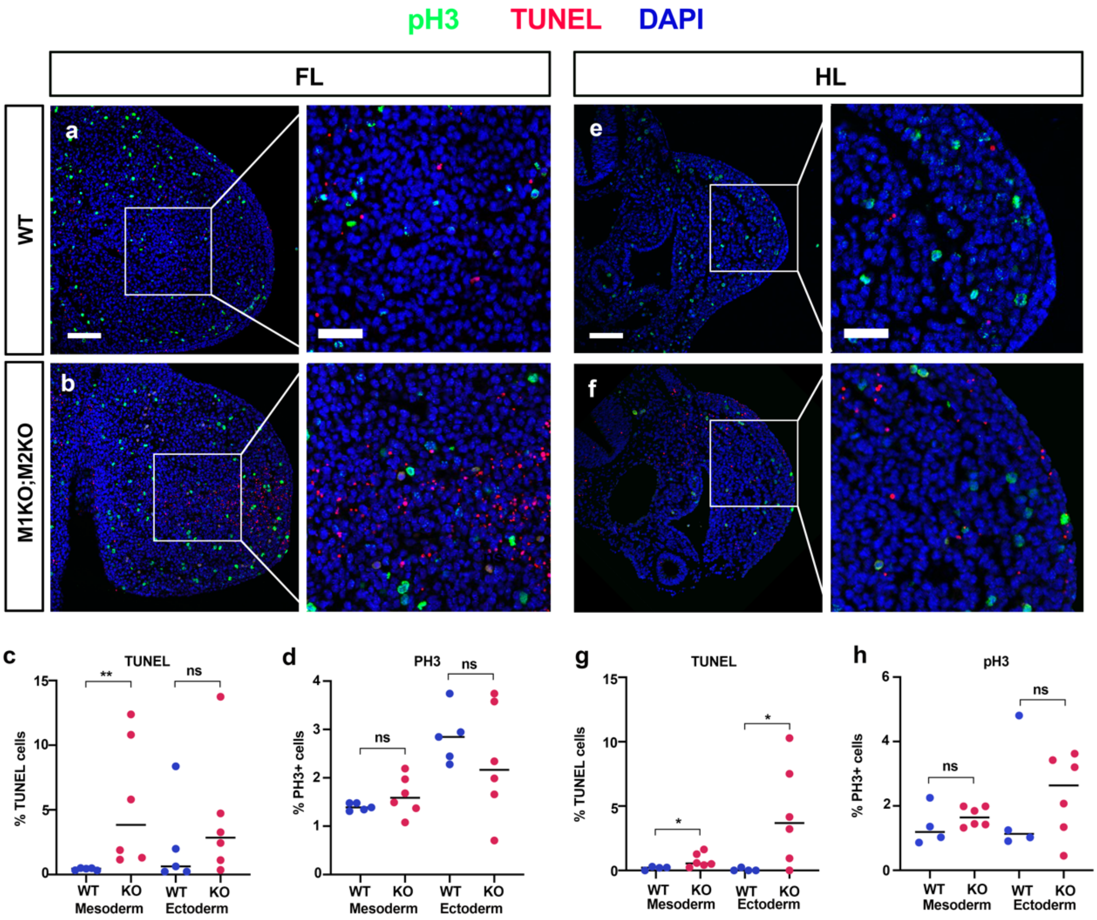

Supplementary Fig 2. Cell death and proliferation in M1KO;M2KO limbs. (a-b) WT and M1KO;M2KO (KO in the graph) E10.5 FL sections and amplified region corresponding to the white square are shown. Scale bars= 100µm and 50µm in the amplified area. Percentage of TUNEL and H3 positive cells is represented in the graphs (c and d respectively) WT FL N=5; KO FL N=6. Exact p-values: TUNEL: 0.0043; 0,2468 and H3: 0,2468; 0,3528. Black bars represent median values (e-h) Sections and graphs of WT and M1KO;M2KO (KO in the graph) HLs. Scale bars= 100µm and 50µm in the amplified area. Statistical comparisons were performed using an unpaired, two-tailed Mann-Whitney test. WT HL N=4; KO HL N=6. Exact p-values from left to right: TUNEL: 0,0190; 0,0190 and pH3: 0,3524; 0,6095. Black bars represent median values. p < 0.05, **p <0.01. Source data are provided as a Source Data file. Source images are available from Mendeley Data[1].

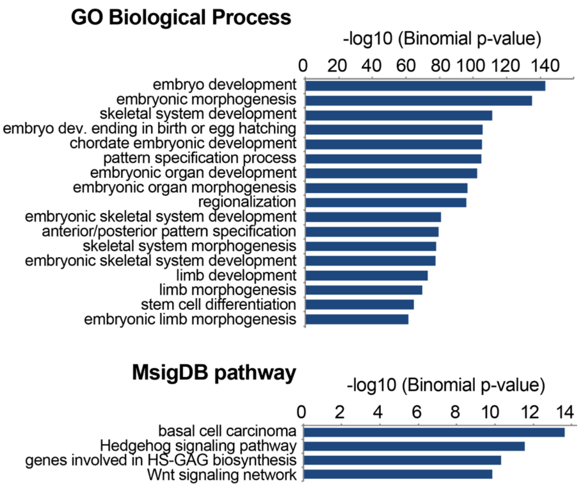

Supplementary Fig 3. Study of gene categories enriched in the Meis-bound gene set. The graphs represent the top over-represented categories from the "Gene Ontology-Biological Process" and "MdsigDB pathway" databases in the Meis-bound gene set (FL-HL common peaks), indicating the p-value of the associations. Statistics: one-tailed Fisher exact test for over-representation of the gene sets explored (hypergeometric test). The p-values are not adjusted for multiple comparisons.

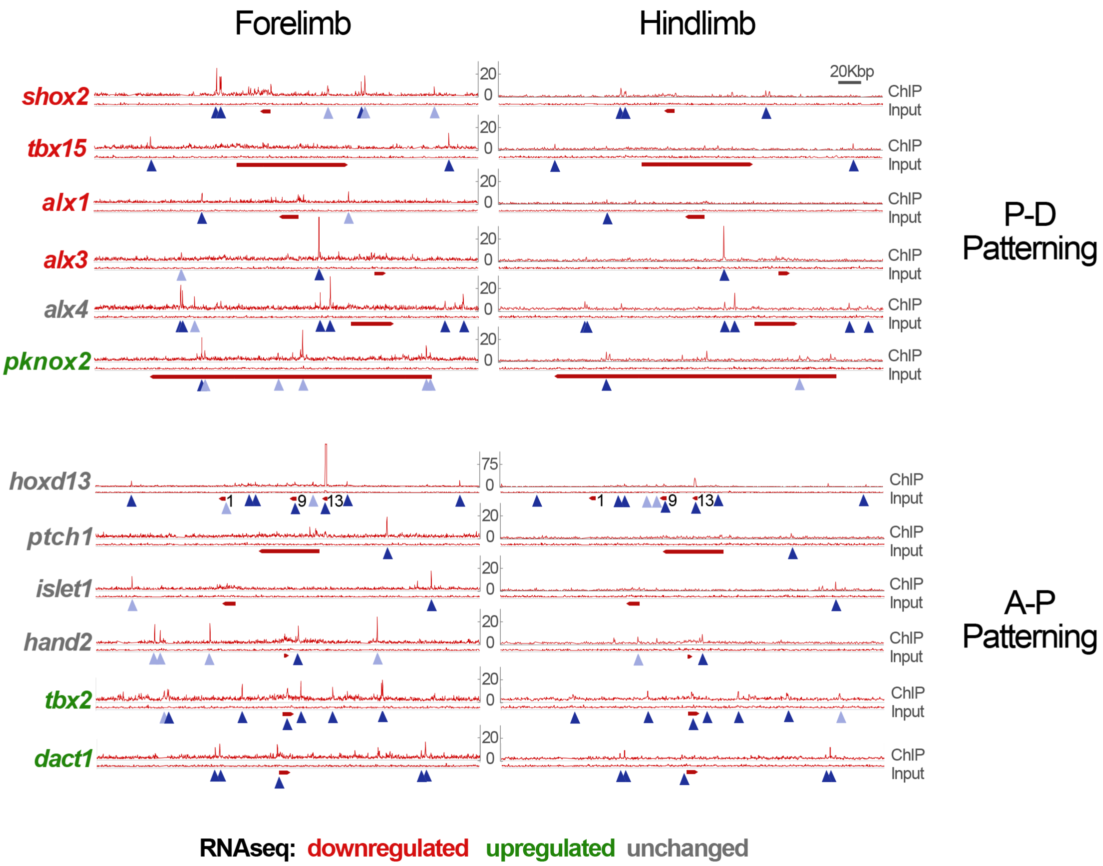

Supplementary Fig 4. Meis ChIPseq binding profile in genes of the PD and AP specification pathways. Meis binding sites in the vicinity of genes of the PD and AP pathways. In red, downregulated genes in the RNAseq and in green, upregulated genes. Dark blue arrowheads mark peaks common to FLs and HLs. Light blue arrowheads mark peaks specific to either FLs or HLs. See "Data availability" section for source data.

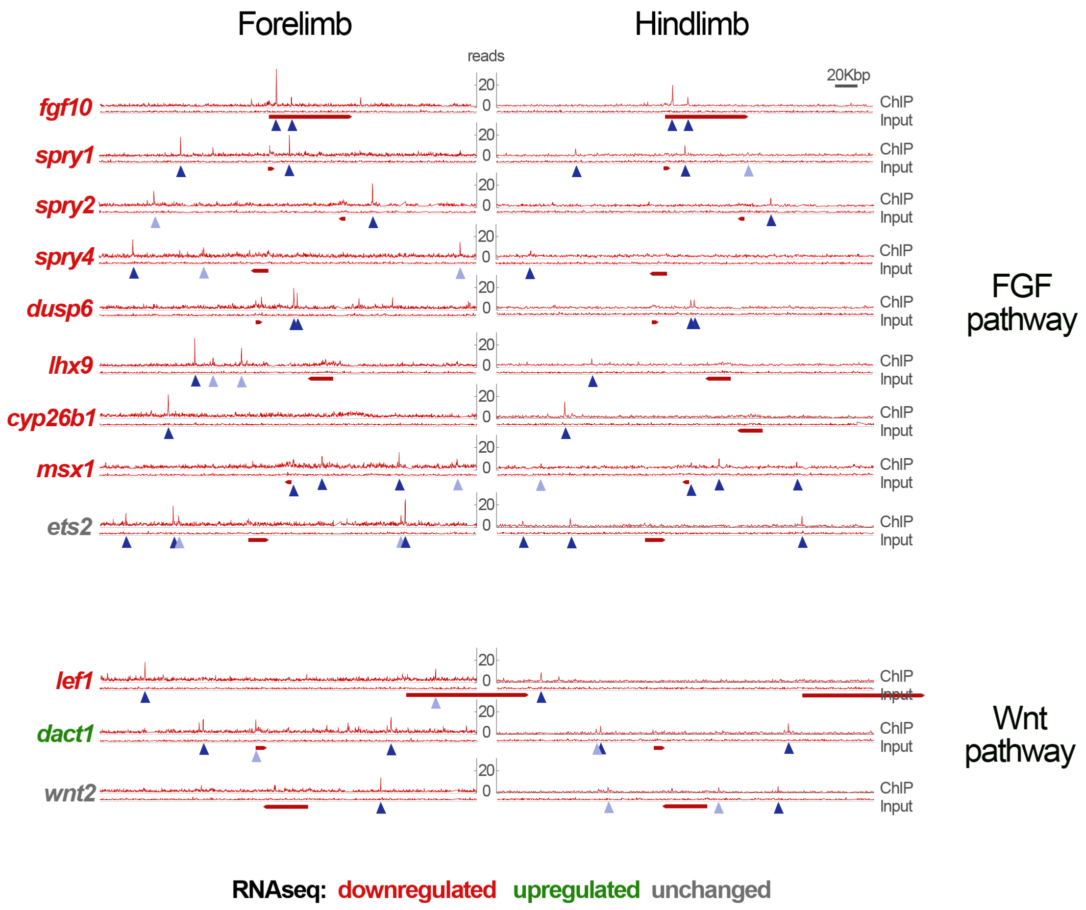

Supplementary Fig 5. Meis ChIPseq binding profile in genes of the Wnt and Fgf pathways. Meis binding sites in the vicinity of genes genes of the Fgf and Wnt pathways. In red, downregulated genes in the RNAseq and in green, upregulated genes. Dark blue arrowheads mark peaks common to FLs and HLs. Light blue arrowheads mark peaks specific to either FLs or HLs. See "Data availability" section for source data.

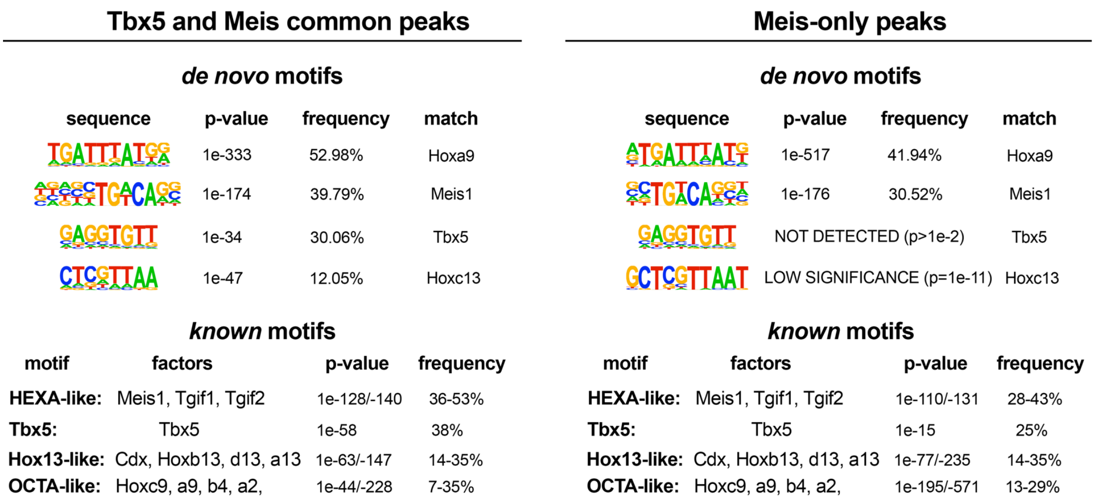

Supplementary Fig 6. Analysis of TBX binding sequences in Meis ChIPseq. HOMER *de novo* and *known* sequence motif identification in Meis and Tbx5 common peaks and in Meis-only peaks.

Supplementary Reference

1. “Mendeley Data” (https://data.mendeley.com/), D.O.I.: 10.17632/r774bxyf8d.1
