# Supplementary material for: Control of mouse limb initiation and antero-posterior patterning by Meis transcription factors
Source: Nat Commun. 2021 May 25;12:3086. doi: 10.1038/s41467-021-23373-9 (PMC8149412; doi:10.1038/s41467-021-23373-9)
Supplement: Supplementary file 2 — Description of Additional Supplementary Files [file 41467_2021_23373_MOESM2_ESM.pdf]

Title: Supplementary Dataset 1.
Description: Summary of RNAseq results comparing wild type with *Meis1* and *Meis2* double knockout limb buds

Title: Supplementary Dataset 2.
Description: Full list of Meis ChIPseq peaks in Fore- and Hindlimb buds

Title: Supplementary Dataset 3.
Description: Genes associated to Meis ChIPseq peaks commonly detected in Fore- and Hindlimb buds.

Title: Supplementary Dataset 4.
Description: Lists of ChIP-seq peaks co-bound by different combinations of Meis, Tbx and Hox transcription factors in limb buds

Title: Supplementary Dataset 5.
Description: Sequences of guide RNAs for CRISPR-Cas9 genomic deletions and sequences of the PCR primers for genotyping and quantitative reverse transcription PCR analysis
