# Supplementary material for: Control of mouse limb initiation and antero-posterior patterning by Meis transcription factors
Source: Nat Commun. 2021 May 25;12:3086. doi: 10.1038/s41467-021-23373-9 (PMC8149412; doi:10.1038/s41467-021-23373-9)
Supplement: Supplementary file 7 — Supplementary Data 5 [file 41467_2021_23373_MOESM7_ESM.docx]

Supplementary dataset 5

Genotyping

| Meis2_Forward | CAAGGACGCAATCTATGGGTA |
| --- | --- |
| Meis2_Reverse | TGCAGAAAACTTTCCTCTTAATCA |

WT band: 422 bp; floxed band: 525 bp

CRISPR/Cas9 sgRNAs

| Fgf10_5’-∆0.4 | GGGCTCCTTGCCTTTAAATCAGG |
| --- | --- |
|  | GAATTAGGTTATAATTGTAGTGG |
| Fgf10_3’-∆0.4 | CTGGCTCTCACAATTAAACATGG |
|  | GAAATGGCTATATGTAGATTTGG |
| Fgf10_14.5∆ | GGGCTCCTTGCCTTTAAATCAGG |
|  | CTGGCTCTCACAATTAAACATGG |
| Hand2_3kb∆ | CCACTGGGTGATCCATAGTATGG |
|  | TATTAGAGCCTCATAATGCATGG |

qPCR

| Fgf10 | Fw CAACTCCGATTTCCACTGATGT Rv GCTGTTCTCCTTCACCAAGT |
| --- | --- |
