# Supplementary material for: Control of mouse limb initiation and antero-posterior patterning by Meis transcription factors
Source: Nat Commun. 2021 May 25;12:3086. doi: 10.1038/s41467-021-23373-9 (PMC8149412; doi:10.1038/s41467-021-23373-9)
Supplement: Supplementary file 9 — Reporting Summary [file 41467_2021_23373_MOESM9_ESM.pdf]

nature research

Corresponding author(s): Miguel Torres

Last updated by author(s): Apr 6, 2021

Reporting Summary

Nature Research wishes to improve the reproducibility of the work that we publish. This form provides structure for consistency and transparency in reporting. For further information on Nature Research policies, see our Editorial Policies and the Editorial Policy Checklist.

Statistics

For all statistical analyses, confirm that the following items are present in the figure legend, table legend, main text, or Methods section.

n/a	Confirmed	
☐	☒	The exact sample size (*n*) for each experimental group/condition, given as a discrete number and unit of measurement
☐	☒	A statement on whether measurements were taken from distinct samples or whether the same sample was measured repeatedly
☐	☒	The statistical test(s) used AND whether they are one- or two-sided<br>*Only common tests should be described solely by name; describe more complex techniques in the Methods section.*
☒	☐	A description of all covariates tested
☒	☐	A description of any assumptions or corrections, such as tests of normality and adjustment for multiple comparisons
☐	☒	A full description of the statistical parameters including central tendency (e.g. means) or other basic estimates (e.g. regression coefficient) AND variation (e.g. standard deviation) or associated estimates of uncertainty (e.g. confidence intervals)
☐	☒	For null hypothesis testing, the test statistic (e.g. *F*, *t*, *r*) with confidence intervals, effect sizes, degrees of freedom and *P* value noted<br>*Give P values as exact values whenever suitable.*
☒	☐	For Bayesian analysis, information on the choice of priors and Markov chain Monte Carlo settings
☒	☐	For hierarchical and complex designs, identification of the appropriate level for tests and full reporting of outcomes
☒	☐	Estimates of effect sizes (e.g. Cohen's *d*, Pearson's *r*), indicating how they were calculated

Our web collection on statistics for biologists contains articles on many of the points above.

Software and code

Policy information about availability of computer code

Data collection	Nikon microscopy camera acquisition software, Illumina Hiseq 2500
Data analysis	ImageJ 2.0, Prism9, MACS2, BWA, GREAT, HOMER, FAStQC, Rsem, TMM, IGV 2.8, Cutadapt, ComBat and Limma (all referenced in the manuscript)

For manuscripts utilizing custom algorithms or software that are central to the research but not yet described in published literature, software must be made available to editors and reviewers. We strongly encourage code deposition in a community repository (e.g. GitHub). See the Nature Research guidelines for submitting code & software for further information.

Data

Policy information about availability of data

All manuscripts must include a data availability statement. This statement should provide the following information, where applicable:
- Accession codes, unique identifiers, or web links for publicly available datasets
- A list of figures that have associated raw data
- A description of any restrictions on data availability

Sequencing data are available at the GEO-NCBI database with accession number GSE134039 for RNAseq and GSE134034 for ChIPseq. Summary tables of the RNAseq and ChIPseq analyses performed are available as supplementary tables 1-4. Numerical source data corresponding to the graphs showing aggregated data in the figures are provided in a "Source Data" excel table. Source images corresponding to all individual specimens analyzed are available from "Mendeley Data" under D.O.I.: 10.17632/r774bxyf8d.1. All non-commercial materials are available upon request.

Field-specific reporting

Please select the one below that is the best fit for your research. If you are not sure, read the appropriate sections before making your selection.

- [x] Life sciences
- [ ] Behavioural & social sciences
- [ ] Ecological, evolutionary & environmental sciences

For a reference copy of the document with all sections, see nature.com/documents/nr-reporting-summary-flat.pdf

Life sciences study design

All studies must disclose on these points even when the disclosure is negative.

Sample size	For qualitative assessments, like determination of gene expression patterns in whole-mounts a minimum of 4 Biological distinct samples (limbs) were used, which is the standard in the field. For quantitative measurements, we used a minimum of 4 Biological distinct samples and a maximum of 13. In our experience this sample size is sufficient to detect possible deviations from the normal morphological or gene expression patterns in developing embryos. There are no publications that specifically describe these practices, however, this sample size calculation is standard in the field.
Data exclusions	No data were excluded
Replication	Biological replicas were at least 4 in each qualitative experiment. The reproducibility for each experiment is detailed in the main text.
Randomization	Allocation was by genotype
Blinding	No blinding was adopted with respect to mutant embryo handling, given that the mutants analyzed show very obvious morphological phenotypes, so it is not possible to keep the experimenter naive with respect to the genotype of the specimens. Nonetheless, genotyping was always performed blinded with respect to the specimen phenotypes, so that the assignment genotype-phenotype was not influenced by the phenotype of the specimens

Reporting for specific materials, systems and methods

We require information from authors about some types of materials, experimental systems and methods used in many studies. Here, indicate whether each material, system or method listed is relevant to your study. If you are not sure if a list item applies to your research, read the appropriate section before selecting a response.

Materials & experimental systems

n/a	Involved in the study	
[ ]	[x]	Antibodies
[x]	[ ]	Eukaryotic cell lines
[x]	[ ]	Palaeontology and archaeology
[ ]	[x]	Animals and other organisms
[x]	[ ]	Human research participants
[x]	[ ]	Clinical data
[x]	[ ]	Dual use research of concern

Methods

n/a	Involved in the study	
[ ]	[x]	ChIP-seq
[x]	[ ]	Flow cytometry
[x]	[ ]	MRI-based neuroimaging

Antibodies

Antibodies used	HoxD9 (H-342 Santa Cruz Biotechnology). anti-phospho-histone 3 antibody (06-570 Sigma-Aldrich). Home-made Meis1 and Meis2, described in Mercader N, Tanaka EM, Torres M. Proximodistal identity during vertebrate limb regeneration is regulated by Meis homeodomain proteins. Development (Cambridge, England) 132, 4131-4142 (2005).
Validation	All functioned as expected, giving the described patterns.Moreover HoxD9 antibody was validated by Western blot (WB) by manufacturer and supported by 4 publications (D'Antò, V. et al. 2006. J. Cell. Biochem. 97: 836-848, Okada, Y. et al. 2003. Blood. 101: 4748-4756, Gattenlohner, S. et al. 2003. Am. J. Pathology. 163: 1081-1090 and Perrais, M. et al. 2001. J. Biol. Chem. 276: 15386-15396). anti-phospho-histone 3 antibody (06-570 Sigma-Aldrich) was validated by WB and immunofluorescence by the manufacturer and supported by several references (such as Function of translationally controlled tumor protein (TCTP) in Eudrilus eugeniae regeneration Subramanian ER, et al. PLoS ONE 12(4), e0175319-e0175319, (2017) and Environmental stresses induce karyotypic instability in colorectal cancer cells Tan Z, et al. Molecular Biology of the Cell 30(1), 42-55, (2019)). Meis1 and 2 was validated in our lab by WB, immunofluorescence and ChIP and suported by several references (such as Mercader N, Tanaka EM, Torres M. Proximodistal identity during vertebrate limb regeneration is regulated by Meis homeodomain proteins. Development (Cambridge, England) 132, 4131-4142 (2005), Delgado et al.,Proximo-distal positional information encoded by an Fgf-regulated gradient of homeodomain transcription factors in the vertebrate limb.,Science Advances,2020, Lopez-Delgado et al, Axial skeleton anterior-posterior patterning is regulated through feedback regulation between Meis transcription factors and retinoic acid, Development 2021).

Animals and other organisms

Policy information about studies involving animals; ARRIVE guidelines recommended for reporting animal research

Laboratory animals	HoxB6CreER, Meis1flox, Meis2 flox, Rosa26lacZ and Fgf10 mutants were all Mus musculus of mixed genetic background. All experimental specimens were non-sexed embryos between 9 and 18 days of development
Wild animals	No wild animals were used in the study
Field-collected samples	No field-collected samples were used in this study
Ethics oversight	Mice were handled in accordance with CNIC Ethics Committee, Spanish laws and the EU Directive 2010/63/EU for the use of animals in research. All mouse experiments were approved by the CNIC and Universidad Autónoma de Madrid Committees for "Ética y Bienestar Animal" and the area of "Protección Animal" of the Community of Madrid

Note that full information on the approval of the study protocol must also be provided in the manuscript.

ChIP-seq

Data deposition

☒ Confirm that both raw and final processed data have been deposited in a public database such as GEO.

☒ Confirm that you have deposited or provided access to graph files (e.g. BED files) for the called peaks.

Data access links<br>*May remain private before publication.*	https://www.ncbi.nlm.nih.gov/geo/query/acc.cgi?acc=GSE134034
Files in database submission	GSM3933626 Meis_Input_FL<br>GSM3933627 Meis_Input_HL<br>GSM3933628 Meis_IP_FL<br>GSM3933629 Meis_IP_HL
Genome browser session<br>(e.g. UCSC)	http://genome.ucsc.edu/s/mtorres%40cnic.es/Meis%20ChIPseq%20E10.5%20buds

Methodology

Replicates	Two experiments were made using , respectively, forelimb buds and hindlimb buds. The degree of overlap was over 80% despite the samples were not identical. More replicas are strongly limited by the fact that small embryonic tissues were used and about 500 embryos were needed to perform these two independent experiments
Sequencing depth	Raw reads: Input_FL: 56591155 ; Input_HL: 65735034 ; IP_FL: 85252035 ; IP_HL: 87665474<br>BWA mapping: Total: Input_FL: 97% ; Input_HL: 96% ; IP_ FL: 83% ; IP_ HL: 88%<br>BWA mapping: Unique mapped sequences excluding Y and mitochondrial chromosomes and unplaced scaffolds (mm9): Input_FL: 43% ; Input_HL: 49% ; IP_FL: 74% ; IP_HL: 66%.
Antibodies	Home-made Meis1 and Meis2, described in Mercader N, Tanaka EM, Torres M. Proximodistal identity during vertebrate limb regeneration is regulated by Meis homeodomain proteins. Development (Cambridge, England) 132, 4131-4142 (2005).
Peak calling parameters	READ MAPPING COMMAND: "bwa aln -t 6 REF.FA SAMPLE.FQ | bwa samse REF.FA - SAMPLE.FQ | samtools view -bSh - | samtools sort - SAMPLE.SORTED.BAM", followd by "MarkDuplicates" and "bamtools filter" to mark duplicates and filter the alignments, respectively.<br>PEAK CALLING COMMAND FOR THE DETECTION OF SHARED PEAKS: "macs2 callpeak -t CHIP_SAMPLE.SORTED.MARKDUP.FILTERED.BED.GZ -c INPUT_SAMPLE.SORTED.MARKDUP.FILTERED.BED.GZ -f BED -g mm -B --call-summits -p 0.022", followed by BEDtools operations to define a consensus set of shared peaks.<br>PEAK CALLING COMMANS FOR THE DETECTION OF SPECIFIC PEAKS: "macs2 callpeak -t CHIP_SAMPLE.SORTED.MARKDUP.FILTERED.BED.GZ -c INPUT_SAMPLE.SORTED.MARKDUP.FILTERED.BED.GZ -f BED -g mm -B --call-summits -p 0.0005", followed by BEDtools operations to define sets of specific peaks, subtracting both the peaks identified in the other condition and the collection of shared peaks identified in the previous step.
Data quality	FastQC was used to perform quality checks on fastq files.<br>The numbers of peaks called by MACS2 that were associated to FDR 5% and above 5-fold enrichment, under the two imposed significance thresholds were 1740 and 831, for FL and HL, respectively, with "-p 0.022", and 1719 and 821, for FL and HL, respectively, with "-p 0.0005".<br>Alignments in BAM format, coverage files in BIGWIG format and peaksets in BED format were visualized with IGV to check the relative intensity and position of peaks at selected locations.
Software	FastQC: Babraham Bioinformatics - FastQC A Quality Control tool for High Throughput Sequence Data [WWW Document], n.d. URL https://www.bioinformatics.babraham.ac.uk/projects/fastqc.<br>CUTADAPT: Martin, M., 2011. Cutadapt removes adapter sequences from high-throughput sequencing reads. EMBnet.journal 17, 10–12.<br>BWA: Li H, Durbin R. Fast and accurate short read alignment with Burrows–Wheeler transform. Bioinformatics 2009;25:1754–60.

MACS2: Zhang, Y., et al, 2008. Model-based Analysis of ChIP-Seq (MACS). Genome Biology 9, R137.
BEDtools: Quinlan, A.R., Hall, I.M., 2010. BEDTools: a flexible suite of utilities for comparing genomic features. Bioinformatics 26, 841–842.
IGV: Robinson, J.T., et al, 2011. Integrative Genomics Viewer. Nat Biotechnol 29, 24–26.
HOMER: Heinz, S., et al, 2010. Simple combinations of lineage-determining transcription factors prime cis-regulatory elements required for macrophage and B cell identities. Mol Cell 38, 576–589.
GREAT: McLean, C.Y., et al, 2010. GREAT improves functional interpretation of cis-regulatory regions. Nat Biotechnol 28, 495–501.
